# Trends and disparities in the prevalence of circulatory disease risk factors among U.S. adults from the National Health Interview Survey database (2019–2022)

**DOI:** 10.1016/j.ijcrp.2025.200393

**Published:** 2025-03-08

**Authors:** Farah Yasmin, Abdul Moeed, Hafsah Alim Ur Rahman, Muhammad Ahmed Ali Fahim, Afia Salman, Maryam Shaharyar, Rohan Kumar Ochani, Afsana Ansari Shaik, Muhammad Sohaib Asghar, M. Chadi Alraies

**Affiliations:** a Yale School of Medicine, New Haven, CT, USA; b Dow University of Health Sciences, Karachi, Pakistan; c Karachi Medical and Dental College, Karachi, Pakistan; d SUNY Upstate Medical University, Syracuse, NY, USA; e Mayo Clinic Rochester, MN, USA; f Advent Health Sebring, FL, USA; g Detroit Medical Center, Detroit, MI, USA

**Keywords:** Circulatory diseases, Risk factors, Prevalence trends, U.S. adults, Diabetes, Obesity, Smoking, Hyperlipidemia, Disparities, NHIS

## Abstract

**Introduction:**

Circulatory diseases are the leading cause of mortality in the United States (U.S)., making it crucial to understand trends and disparities in the prevalence of cardiovascular risk factors including diabetes, obesity, smoking, and hyperlipidemia.

**Methods:**

Data from the Centers for Disease Control and Prevention (CDC)'s National Health Interview Survey (NHIS) database was analyzed for adults aged 18 and older from 2019 to 2022. Prevalence percentages and Annual Percentage Changes (APCs) were calculated using regression analysis with Joinpoint, with 95 % confidence intervals (CI). The data was stratified by year, gender, age, race, nativity, veteran status, social vulnerability, employment status, and geographic distribution.

**Results:**

Among circulatory disease risk factors, obesity had the highest prevalence remaining consistent across all years. The highest obesity rates were observed amongst females, those aged 45–64, and Black or African American adults, with regional peaks in the South and Midwest. High Cholesterol, the second most prevalent risk factor, rose significantly from 20.1 % to 22 % [APC: 3.3175∗ (95 % CI: 1.1417 to 5.5416)] with males [APC: 3.3175∗ (95 % CI: 1.1417 to 5.5416)] and females [APC: 3.1315∗ (95 % CI: 3.0191 to 3.2428)] both showing significant increases over time. Furthermore, those aged >65 yrs and White adults in addition to those residing in the Northeast and South revealed the highest rates. Smoking rates remained steady, with a higher male prevalence which showed a significant decrease [APC: −5.0336∗ (95 % CI: −9.156 to −0.6731)] over time. Diabetes prevalence was stable, with males, adults aged 64 and above, American Indians and Black or African American adults and those residing in the southern region consistently showing the highest rates of incidence.

**Conclusion:**

Significant disparities and increasing trends in risk factors for circulatory diseases have been identified, highlighting the need for targeted interventions, particularly for high-risk groups such as males, older adults, veterans, and the unemployed.

## 1 Introduction

Circulatory also termed as cardiovascular diseases encompass all conditions or disorders affecting the heart and the blood vessels including coronary artery disease, hypertension and myocardial infarction [1]. Circulatory disorders and other non-communicable diseases constitute a major age-standardized global burden of disease across the globe, with ischemic heart disease being the leading cause of global circulatory disease burden [2]. Despite the decline in the cardiovascular disease death rates in the United States (U.S), cardiovascular disease remains the leading cause of mortality among the U.S adult population [3]. Some of the known risk factors for circulatory diseases constitute diabetes mellitus, alcohol consumption, smoking, hypertension, and abdominal obesity [4,5]. The decline in death rates from cardiovascular diseases can be explained by improvements in medical care and subsequent primary prevention aimed at the modification of physiological risk factors [6]. However, the stagnated decline in the mortality rates of cardiovascular diseases is attributed to a greater burden of risk factors and disability, particularly in young adults [7,8].

In accordance with the U.S. National Health and Nutrition Examination Survey data, the age-adjusted prevalence of hypertension was approximately 44.7 % during 2017–2020 [9]. Another recent study reported increasing trends in the prevalence of blood pressure and obesity from the period 1999–2012 to 2015–2020. Limited control of cardiometabolic risk factors in certain comorbidities such as diabetes contributes to a greater prevalence of obesity, which is estimated to be 46.9 % and 58.1 % in the years 1999–2002 and 2015–2020, respectively [10]. Tobacco consumption is labeled the fifth leading cause of cardiovascular mortality, responsible for more than three million cardiovascular deaths overall in the year 2021 [11].

While the existing literature provides data on the prevalence of circulatory disease risk factors, there is a paucity of research and latest data regarding the associated trends and disparities in these risk factors among U.S. adults. The identification of risk factors and the associated trends and disparities in these risk factors are integral to the management and prevention of circulatory diseases and the facilitation of strategic development to mitigate the risk of adverse complications and deaths [4]. In this study, we aimed to assess the trends and disparities in the prevalence of circulatory disease risk factors in the U.S. adult population between 2019 and 2022.

## 2 Methods

Our study focused on the prevalence of four major risk factors of circulatory diseases i.e., obesity, elevated cholesterol levels, current cigarette smoking, and diagnosed diabetes mellitus among U.S. adults aged 18 and older utilizing data from the Centers for Disease Control (CDC)'s National Center for Health Statistics (NCHS) Interactive Summary Health Statistics for Adults conducted by the CDC NCHS which uses home-based interviews and clustered sampling to select dwellings with telephone-only interviews being used during the coronavirus disease 2019 (COVID-19) pandemic [12]. Institutional Review Board approval was not required for this study as all data utilized was publicly available.

### 2.1 Definitions and data abstraction

To diagnose high cholesterol, respondents were initially asked if a doctor or other health professional had ever told them that they had high cholesterol. If yes, they were then asked if they had been told by a doctor or other health professional that they had high cholesterol during the past 12 months and if they were taking prescribed cholesterol-lowering medication. Respondents answering yes to either question were classified as having high cholesterol.

Obesity was defined as having a body mass index (BMI) of ≥30.0 kg/m^2^. This was calculated from information obtained in response to survey questions pertaining to height and weight. To classify individuals as having diagnosed diabetes, participants were asked if a doctor or other health professional had ever told them that they had diabetes. Responses of prediabetes, borderline diabetes or gestational diabetes were excluded. Initially, respondents were asked if they smoked ≥100 cigarettes in their entire lives, and if so, do they now smoke every day, some days, or not at all. Current cigarette smokers were classified as those who smoked every day or some days. Additionally, trends for arterial hypertension among U.S. adults were presented in Supplementary Fig. 1.

The data was stratified by year, gender, age, race, nativity, veteran status, employment status, geographical region, social vulnerability index (SVI), and metropolitan statistical area (MSA) using survey responses. SVI assesses the vulnerability of census tracts/counties using U.S. census data across four themes: socioeconomic status, household composition, race/language status, and housing/transportation. SVI expresses the relative vulnerability as a percentile ranking ranging from 0 to 1 and is categorized into "little to no social vulnerability” (0–0.2500) "low social vulnerability (0.2501–0.500) medium social vulnerability (0.5001–0.7500) high social vulnerability (0.7501–1) [13]. The demographic variables used were based on information obtained from the household roster, sample adult, and sample child components of the NHIS [14].

### 2.2 Statistical analysis

We provided prevalence percentages with 95 % confidence intervals (CI) and employed Joinpoint regression analysis to calculate the annual percentage change (APC) of prevalence, determining statistical significance with 95 % CI.

## 3 Results

### 3.1 Annual trends and disparities in the prevalence of diagnosed diabetes mellitus among U.S. Adults

**Fig. 1:**
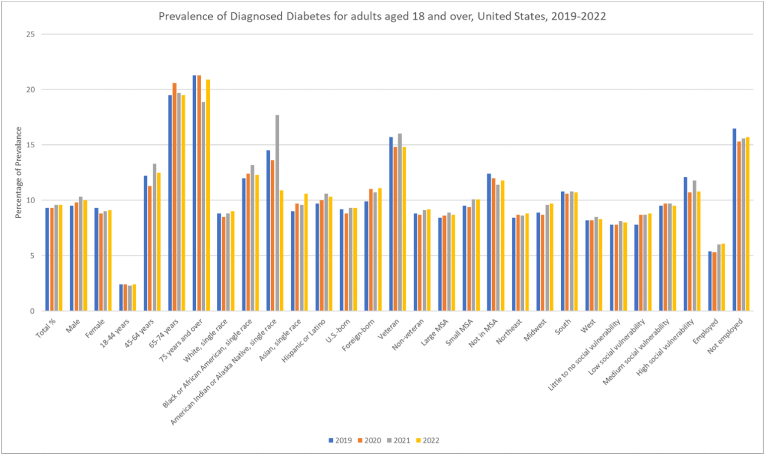
Prevalence of Diagnosed Diabetes for adults 18 and over in the United States 2019–2022.

**Fig. 2:**
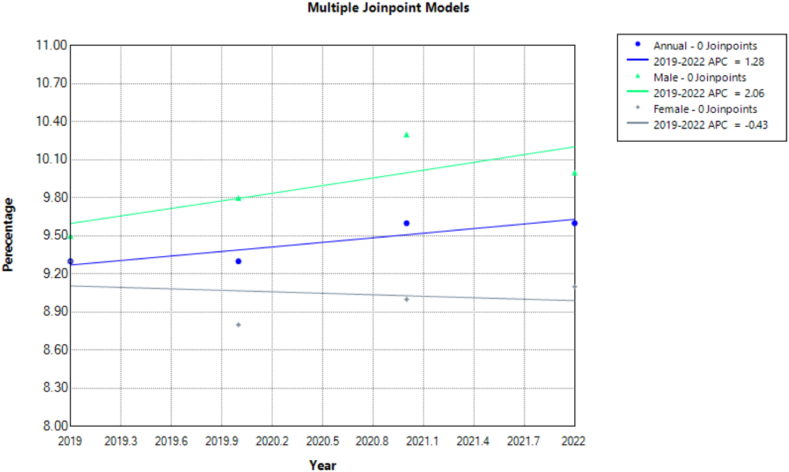
Annual Percentage Change of Diagnosed Diabetes for adults 18 and over in the United States 2019–2022.

The prevalence of diagnosed diabetes among U.S. adults aged 18 and over remained steady at around 9.3 (95 % CI: 9.0, 9.7) in 2019 and 2020, with a slight increase to 9.6 (95 % CI: 9.2, 10.0) in 2021 and 2022. However, these findings were nonsignificant on annual trend analysis with an APC of 1.278 (95 % CI: −0.296 to 2.8866) from 2019 to 2022. Males consistently showed higher diabetes prevalence than females, rising from 9.5 (95 % CI: 8.9, 10.0) in 2019 to 10 (95 % CI: 9.5, 10.6) in 2022. Contrastingly, female prevalence lowered from 9.3 (95 % CI: 8.7, 9.8) in 2019 to 9.1 (95 % CI: 8.6, 9.6) in 2022. However, these changes were insignificant for both genders [male APC: 2.0573 (95 % CI: −0.9107 to 5.0808); female APC: −0.4266 (95 % CI: −3.0439 to 2.2794]. Diagnosed diabetes showed higher rates in those aged 65–74 years and 75 years with 21.3 (95 % CI: 19.7, 22.9) for those 75 and older in 2019 and 2020 (Table 1, Fig. 1, Fig. 2, Supplementary Table 1).

**Table 1:** Prevalence percentage of Diagnosed Diabetes, High Cholesterol, Cigarette Smoking and Obesity for adults aged 18 and over, United States, 2019–2022. *CDC: Centers for Disease Control; MSA: Metropolitan Statistical Area*.

	Diagnosed Diabetes				Obesity				Cigarette smoking				High Cholesterol			
**Year**	2019	2020	2021	2022	2019	2020	2021	2022	2019	2020	2021	2022	2019	2020	2021	2022
**Total %**	9.3 (9.0, 9.7)	9.3 (8.9, 9.7)	9.6 (9.2, 10.0)	9.6 (9.2, 10.0)	32.1 (31.4, 32.8)	33 (32.3, 33.8)	32.8 (32.1, 33.6)	33.1 (32.4, 33.9)	14 (13.5, 14.5)	12.5 (11.9, 13.0)	11.5 (11.1, 12.0)	11.6 (11.1, 12.1)	20.1 (19.6, 20.6)	20.8 (20.3, 21.4)	21.6 (21.1, 22.2)	22 (21.5, 22.6)
**Male**	9.5 (8.9, 10.0)	9.8 (9.3, 10.4)	10.3 (9.7, 10.9)	10 (9.5, 10.6)	32.1 (31.1, 33.1)	32.7 (31.7, 33.6)	32.6 (31.6, 33.6)	32.8 (31.8, 33.8)	15.3 (14.6, 16.1)	14.1 (13.3, 14.9)	13.1 (12.4, 13.8)	13.2 (12.5, 14.0)	20.7 (19.9, 21.4)	21.6 (20.8, 22.4)	22.7 (21.9, 23.5)	22.7 (21.8, 23.5)
**Female**	9.3 (8.7, 9.8)	8.8 (8.3, 9.3)	9 (8.5, 9.6)	9.1 (8.6, 9.6)	32.1 (31.2, 33.1)	33.4 (32.4, 34.4)	33 (32.0, 34.0)	33.5 (32.5, 34.5)	12.7 (12.1, 13.4)	11 (10.3, 11.6)	10.1 (9.5, 10.6)	10 (9.4, 10.7)	19.5 (18.8, 20.2)	20.1 (19.4, 20.8)	20.7 (19.9, 21.4)	21.4 (20.7, 22.2)
**18**–**44 years**	2.4 (2.1, 2.8)	2.4 (2.1, 2.8)	2.3 (2.0, 2.7)	2.4 (2.1, 2.8)	30.3 (29.2, 31.4)	31.7 (30.5, 32.9)	31.1 (30.0, 32.2)	31.1 (30.0, 32.3)	14.4 (13.7, 15.2)	12.4 (11.6, 13.3)	10.8 (10.1, 11.5)	10.6 (9.9, 11.3)	4.7 (4.3, 5.2)	5.1 (4.6, 5.6)	5.4 (4.9, 5.9)	5.7 (5.2, 6.2)
**45**–**64 years**	12.2 (11.4, 13.0)	11.3 (10.5, 12.1)	13.3 (12.5, 14.1)	12.5 (11.7, 13.3)	36.8 (35.6, 38.0)	37.2 (36.0, 38.4)	38.3 (37.1, 39.5)	38 (36.8, 39.2)	17 (16.1, 17.9)	14.9 (14.0, 15.9)	14.9 (14.1, 15.8)	15.1 (14.2, 16.0)	25.5 (24.5, 26.5)	26.4 (25.3, 27.5)	27.7 (26.7, 28.8)	27.5 (26.4, 28.6)
**65**–**74 years**	19.5 (18.2, 20.8)	20.6 (19.2, 21.9)	19.7 (18.4, 21.0)	19.5 (18.2, 20.9)	33.4 (31.9, 35.0)	34.8 (33.2, 36.4)	33 (31.4, 34.6)	34.1 (32.5, 35.7)	10.6 (9.6, 11.7)	11.5 (10.4, 12.7)	10.6 (9.6, 11.6)	11.5 (10.5, 12.6)	44.8 (43.2, 46.5)	45 (43.4, 46.5)	46.6 (44.9, 48.3)	47.4 (45.8, 49.0)
**75 years and over**	21.3 (19.7, 22.9)	21.3 (19.7, 23.0)	18.9 (17.4, 20.5)	20.9 (19.3, 22.5)	22.6 (21.0, 24.3)	22.3 (20.8, 23.9)	21.8 (20.2, 23.4)	24.9 (23.2, 26.6)	4.7 (3.9, 5.7)	5.2 (4.3, 6.2)	4.8 (4.1, 5.6)	4.3 (3.6, 5.1)	46.5 (44.7, 48.4)	47.3 (45.4, 49.2)	46.4 (44.4, 48.4)	49.1 (47.3, 51.0)
**White, single race**	8.8 (8.4, 9.3)	8.5 (8.1, 8.9)	8.8 (8.4, 9.3)	9 (8.5, 9.4)	31.9 (31.1, 32.6)	32.3 (31.6, 33.1)	32.3 (31.4, 33.1)	32.6 (31.8, 33.5)	14.6 (14.1, 15.2)	12.6 (12.0, 13.2)	12.2 (11.7, 12.7)	12.2 (11.6, 12.7)	21.3 (20.7, 21.9)	22.2 (21.6, 22.9)	23 (22.3, 23.7)	23.6 (22.9, 24.3)
**Black or African American, single race**	12 (10.8, 13.3)	12.4 (11.1, 13.9)	13.2 (11.8, 14.7)	12.3 (11.1, 13.5)	40.6 (38.5, 42.7)	44.5 (42.3, 46.8)	44 (41.8, 46.1)	42.8 (40.9, 44.7)	14.8 (13.3, 16.4)	14.2 (12.5, 16.0)	11.6 (10.3, 13.1)	13.9 (12.5, 15.4)	18.3 (16.8, 19.8)	18.6 (17.0, 20.3)	18.9 (17.4, 20.5)	18.1 (16.7, 19.6)
**American Indian or Alaska Native, single race**	14.5 (10.3, 19.7)	13.6 (8.5, 20.3)	17.7 (13.0, 23.2)	10.9 (7.4, 15.2)	38.4 (31.3, 45.9)	41.7 (33.2, 50.5)	42.2 (28.1, 57.2)	39.1 (31.6, 47.0)	18.2 (10.5, 28.5)	25.3 (17.6, 34.3)	16.5 (8.3, 28.2)	16.9 (10.5, 25.2)	14.2 (9.9, 19.5)	16.5 (11.6, 22.4)	18.1 (12.7, 24.8)	17.8 (13.4, 22.9)
**Asian, single race**	9 (7.3, 11.0)	9.7 (7.9, 11.7)	9.6 (8.1, 11.4)	10.6 (8.8, 12.6)	10.3 (8.5, 12.2)	10.2 (8.4, 12.2)	11 (9.4, 12.9)	12.6 (10.8, 14.5)	7.2 (5.6, 9.0)	7.9 (6.3, 9.8)	5.4 (4.1, 7.0)	4.6 (3.5, 6.0)	17 (15.0, 19.1)	19.7 (17.4, 22.3)	20.4 (18.3, 22.6)	21.1 (18.9, 23.5)
**Hispanic or Latino**	9.7 (8.5, 10.9)	10 (8.8, 11.3)	10.6 (9.5, 11.7)	10.3 (9.2, 11.5)	35.8 (33.9, 37.6)	38 (35.9, 40.2)	37 (35.2, 38.9)	37.6 (35.7, 39.4)	8.8 (7.8, 9.9)	8 (7.0, 9.2)	7.7 (6.8, 8.7)	8 (7.1, 8.9)	16.2 (14.9, 17.6)	16.2 (14.8, 17.7)	16.4 (15.1, 17.7)	16.6 (15.3, 17.9)
**U.S.-born**	9.2 (8.8, 9.6)	8.8 (8.4, 9.2)	9.3 (8.9, 9.8)	9.3 (8.9, 9.7)	34 (33.2, 34.8)	34.7 (33.9, 35.5)	34.7 (33.9, 35.6)	35.1 (34.3, 35.9)	15.4 (14.8, 16.0)	13.5 (12.9, 14.1)	12.6 (12.1, 13.1)	12.6 (12.1, 13.2)	20.5 (19.9, 21.1)	21.1 (20.5, 21.7)	22 (21.3, 22.6)	22.6 (22.0, 23.2)
**Foreign-born**	9.9 (8.9, 11.0)	11 (9.9, 12.2)	10.7 (9.7, 11.9)	11.1 (10.0, 12.2)	24.5 (22.9, 26.2)	25.8 (24.1, 27.6)	24.4 (22.9, 26.0)	25.1 (23.5, 26.7)	7.9 (7.0, 8.9)	7.7 (6.7, 8.7)	6.8 (5.9, 7.7)	6.9 (6.0, 7.8)	19.2 (17.9, 20.6)	19.7 (18.3, 21.3)	20.6 (19.2, 22.0)	20.7 (19.4, 22.2)
**Veteran**	15.7 (14.2, 17.3)	14.8 (13.4, 16.2)	16 (14.3, 17.7)	14.8 (13.1, 16.6)	34.9 (32.9, 36.9)	36.3 (34.1, 38.6)	34 (31.8, 36.3)	35.8 (33.6, 38.2)	17.3 (15.5, 19.1)	14.5 (12.7, 16.5)	14.3 (12.7, 16.0)	13.5 (11.9, 15.2)	34.8 (32.7, 36.9)	35.2 (33.2, 37.3)	39.1 (36.9, 41.3)	37.5 (35.4, 39.7)
**Non-veteran**	8.8 (8.4, 9.2)	8.7 (8.2, 9.1)	9.1 (8.7, 9.5)	9.2 (8.8, 9.6)	32 (31.3, 32.7)	32.8 (32.0, 33.5)	32.7 (32.0, 33.5)	33 (32.2, 33.8)	13.7 (13.2, 14.2)	12.2 (11.7, 12.8)	11.3 (10.8, 11.8)	11.4 (10.9, 11.9)	18.9 (18.4, 19.4)	19.6 (19.0, 20.2)	20.3 (19.7, 20.9)	21 (20.4, 21.6)
**Large MSA**	8.4 (7.9, 8.9)	8.6 (8.0, 9.1)	8.9 (8.4, 9.4)	8.7 (8.2, 9.2)	28.8 (27.9, 29.8)	30.3 (29.3, 31.3)	29.3 (28.4, 30.2)	30.5 (29.6, 31.4)	12 (11.4, 12.7)	10.3 (9.7, 11.0)	8.8 (8.2, 9.3)	9.2 (8.6, 9.8)	18.7 (18.0, 19.4)	19.9 (19.1, 20.6)	20.3 (19.6, 21.1)	20.6 (19.9, 21.4)
**Small MSA**	9.5 (8.9, 10.2)	9.4 (8.7, 10.0)	10.1 (9.4, 10.9)	10.1 (9.4, 10.9)	34.4 (33.2, 35.7)	35 (33.6, 36.4)	35.7 (34.3, 37.2)	34.9 (33.4, 36.3)	14.9 (14.0, 15.9)	13.3 (12.3, 14.4)	13.7 (12.8, 14.6)	13 (12.1, 14.0)	20.6 (19.6, 21.5)	21.3 (20.2, 22.4)	22.7 (21.5, 23.8)	22.5 (21.4, 23.6)
**Not in MSA**	12.4 (11.2, 13.7)	12 (10.7, 13.4)	11.4 (10.2, 12.8)	11.8 (10.8, 12.9)	39.7 (37.8, 41.7)	39.8 (37.6, 42.0)	40.4 (38.0, 42.9)	40.2 (38.1, 42.3)	19.4 (18.0, 20.9)	19 (17.3, 20.9)	18 (16.5, 19.7)	18.1 (16.4, 20.0)	24.2 (22.7, 25.8)	23.6 (22.0, 25.2)	24.7 (22.9, 26.6)	26.8 (25.2, 28.4)
**Northeast**	8.4 (7.5, 9.3)	8.7 (7.8, 9.6)	8.6 (7.7, 9.6)	8.8 (8.0, 9.7)	29.6 (28.1, 31.1)	29.9 (28.2, 31.6)	28.5 (26.9, 30.1)	29.6 (27.7, 31.5)	12.8 (11.6, 14.0)	10.4 (9.3, 11.5)	10.4 (9.3, 11.6)	9.9 (8.9, 11.0)	21.1 (19.9, 22.4)	22.1 (20.7, 23.5)	22.2 (20.9, 23.6)	23.1 (21.7, 24.5)
**Midwest**	8.9 (8.1, 9.7)	8.7 (7.8, 9.6)	9.6 (8.7, 10.5)	9.7 (8.9, 10.5)	33.6 (31.9, 35.3)	34.7 (33.2, 36.2)	35.3 (33.8, 36.8)	35.3 (33.7, 36.9)	16.4 (15.2, 17.6)	15.2 (14.0, 16.5)	14 (13.0, 15.1)	13.6 (12.5, 14.7)	20.4 (19.3, 21.5)	20.7 (19.6, 21.8)	21.5 (20.4, 22.7)	22.2 (21.0, 23.4)
**South**	10.8 (10.1, 11.4)	10.6 (9.9, 11.3)	10.8 (10.1, 11.5)	10.7 (10.0, 11.4)	34.5 (33.3, 35.7)	36.8 (35.6, 38.0)	35.1 (33.8, 36.4)	35.6 (34.4, 36.8)	15.4 (14.6, 16.3)	14.1 (13.1, 15.1)	12.4 (11.6, 13.2)	13.4 (12.5, 14.2)	20.9 (20.1, 21.9)	22.4 (21.4, 23.4)	23.2 (22.2, 24.2)	23 (22.0, 24.0)
**West**	8.2 (7.5, 9.0)	8.2 (7.5, 9.1)	8.5 (7.7, 9.3)	8.3 (7.5, 9.1)	29 (27.6, 30.4)	28 (26.6, 29.3)	30.1 (28.6, 31.8)	30 (28.6, 31.3)	10.4 (9.5, 11.3)	9 (8.3, 9.7)	8.9 (8.1, 9.7)	8.2 (7.3, 9.2)	17.6 (16.6, 18.7)	17.5 (16.5, 18.5)	18.8 (17.7, 20.0)	19.7 (18.6, 20.8)
**Little to no social vulnerability**	7.8 (7.0, 8.5)	7.8 (6.9, 8.6)	8.1 (7.3, 9.0)	8 (7.1, 8.9)	29.7 (28.2, 31.3)	30.4 (28.8, 32.0)	29.5 (28.0, 31.1)	31.4 (29.5, 33.2)	13.1 (11.9, 14.4)	10.5 (9.5, 11.7)	10.2 (9.2, 11.3)	12.1 (10.8, 13.4)	20.3 (19.2, 21.4)	20.4 (19.1, 21.7)	21.5 (20.3, 22.8)	22 (20.6, 23.5)
**Low social vulnerability**	7.8 (7.1, 8.6)	8.7 (7.9, 9.5)	8.7 (8.0, 9.5)	8.8 (8.1, 9.6)	30.4 (29.1, 31.8)	31.5 (30.1, 33.0)	31 (29.6, 32.5)	32.7 (31.3, 34.2)	13.6 (12.6, 14.5)	11.8 (10.8, 12.8)	11.1 (10.1, 12.1)	10.3 (9.4, 11.3)	19 (18.0, 20.0)	20.6 (19.4, 21.7)	21.6 (20.4, 22.7)	22.4 (21.3, 23.5)
**Medium social vulnerability**	9.5 (8.8, 10.2)	9.7 (9.0, 10.4)	9.7 (9.1, 10.4)	9.5 (8.8, 10.2)	32.8 (31.5, 34.1)	34 (32.7, 35.3)	33 (31.7, 34.3)	33.3 (31.9, 34.7)	15 (14.0, 15.9)	13.6 (12.6, 14.6)	12.6 (11.7, 13.5)	12.2 (11.2, 13.2)	20.1 (19.1, 21.1)	21.3 (20.3, 22.4)	21.8 (20.8, 22.9)	23.1 (22.0, 24.3)
**High social vulnerability**	12.1 (11.2, 13.1)	10.7 (9.7, 11.6)	11.8 (10.8, 12.8)	10.8 (10.0, 11.5)	35 (33.5, 36.5)	35.6 (34.0, 37.3)	37.4 (35.6, 39.2)	34.1 (32.8, 35.4)	13.8 (12.7, 15.1)	13.2 (11.9, 14.6)	11.7 (10.7, 12.8)	11.8 (10.9, 12.7)	21.2 (20.0, 22.4)	20.8 (19.6, 22.1)	21.6 (20.3, 22.9)	20.9 (19.9, 22.0)
**Employed**	5.4 (5.0, 5.8)	5.3 (4.9, 5.7)	6 (5.6, 6.4)	6.1 (5.6, 6.5)	32.7 (31.8, 33.6)	33 (32.1, 33.9)	33.1 (32.2, 34.1)	33.5 (32.5, 34.4)	13.8 (13.2, 14.4)	11.7 (11.0, 12.3)	10.9 (10.4, 11.5)	10.5 (10.0, 11.1)	13.6 (13.0, 14.2)	14 (13.4, 14.6)	14.9 (14.2, 15.5)	15.3 (14.7, 16.0)
**Not employed**	16.5 (15.7, 17.3)	15.3 (14.5, 16.1)	15.6 (14.8, 16.3)	15.7 (14.9, 16.5)	31.4 (30.4, 32.5)	33.2 (32.1, 34.3)	32.5 (31.4, 33.7)	32.9 (31.8, 34.1)	14.3 (13.5, 15.1)	13.7 (12.8, 14.6)	12.5 (11.8, 13.3)	13.3 (12.4, 14.1)	32.3 (31.2, 33.3)	31.9 (30.8, 33.0)	33.1 (32.0, 34.2)	34.3 (33.3, 35.4)

White adults consistently had the lowest prevalence rates ranging from 8.8 (95 % CI: 8.4, 9.3) to 9 (95 % CI: 8.5, 9.4) throughout the study period. American Indian or Alaska Natives and Black African Americans showed the highest prevalence rates with 14.5 (95 % CI: 10.3, 19.7) in 2019 which increased to 17.7 (95 % CI: 13.0, 23.2) in 2021 followed by a decrease to 10.9 (95 % CI: 7.4, 15.2) in 2022, however, this trend was observed to be insignificant. Asian adults witnessed a statistically significant increase from 9 (95 % CI: 7.3, 11.0) in 2019 to 10.6 (95 % CI: 8.8, 12.6) in 2022.

Non-MSA areas showed the highest prevalence, decreasing from 12.4 (95 % CI: 11.2, 13.7) in 2019 to 11.8 (95 % CI: 10.8, 12.9) in 2022 while large MSA had the lowest rates. MSAs did not correlate with changes in diabetes prevalence over the years on statistical investigation. In census regions, the West had the lowest prevalence with the South having the highest both remaining relatively stable from 8.2 (95 % CI: 7.5, 9.0) to 8.3 (95 % CI: 7.5, 9.1) and 10.8 (95 % CI: 10.1, 11.4) to 10.7 (95 % CI: 10.0, 11.4) throughout the four years analyzed respectively. The Midwest showed a significant increase in prevalence on annual trend analysis.

Increasing social vulnerability correlated with higher diabetes rates with the most vulnerable group showing a decline in incidence from 12.1 (95 % CI: 11.2, 13.1) to 10.8 (95 % CI: 10.0, 11.5). All trends related to social vulnerability were insignificant on analysis. Unemployed individuals generally had triple the rates than the employed, with a gap decreasing from 16.5 (95 % CI: 15.7, 17.3) in 2019 to 15.7 (95 % CI: 14.9, 16.5) in 2022, versus 5.4 (95 % CI: 5.0, 5.8) to 6.1 (95 % CI: 5.6, 6.5) for the employed. Trend in diagnosed diabetes gained significance for employed individuals only. Foreign-born individuals reported consistently higher prevalence rates compared to U.S.-born. Veterans had substantially higher prevalence rates than non-veterans though it decreased insignificantly from 15.7 (95 % CI: 14.2, 17.3) in 2019 to 14.8 (95 % CI: 13.1, 16.6) in 2022, still higher than the significant increase from 8.8 (95 % CI: 8.4, 9.2) to 9.2 (95 % CI: 8.8, 9.6) for non-veterans.

### 3.2 Annual trends and disparities in the prevalence of obesity among U.S. Adults

The prevalence of obesity stayed relatively constant at around 32.1 (95 % CI: 31.1, 33.1) in 2019 to 32.8 (95 % CI: 31.8, 33.8) in 2022 showing insignificant changes on analysis [APC: 0.8632 (95 % CI: −0.0334 to 1.7804]. Both genders had comparable prevalence rates of obesity at 32.1 (95 % CI: 31.1, 33.1) in 2019 increasing significantly across the study period to 32.8 (95 % CI: 31.8, 33.8) for males [male APC: 0.6185∗ (95 % CI: 0.0422 to 1.2038)] and insignificantly to 33.5 (95 % CI: 32.5, 34.5) for females [female APC: 1.167 (95 % CI: −0.2284 to 2.5773)].

**Fig. 3:**
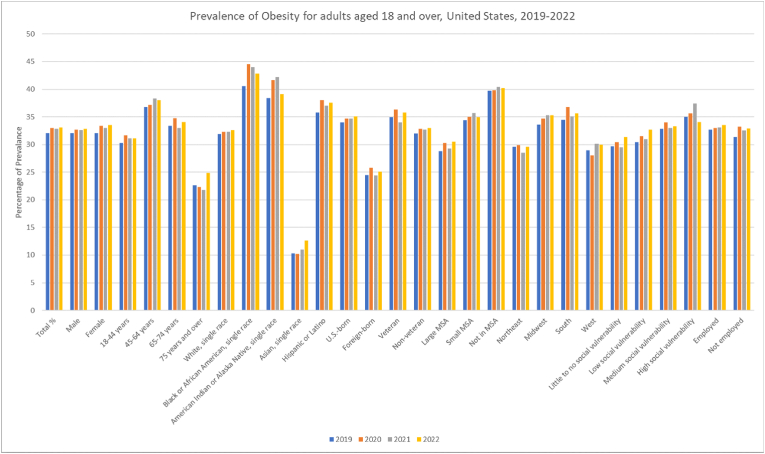
Prevalence of Obesity for adults 18 and over in the United States 2019–2022.

**Fig. 4:**
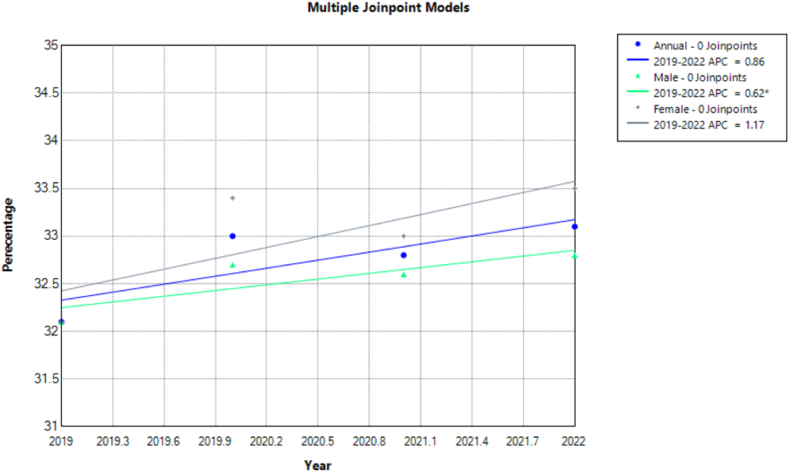
Annual Percentage Change of Obesity for adults 18 and over in the United States 2019–2022.

Age-specific trends revealed patients aged 45–64 years to have higher rates of obesity at 38.3 (95 % CI: 37.1, 39.5) in 2021, and the trend was noted to have significantly increased over the years. However, those aged 75 years and above had the lowest rates among all age groups from 22.6 (95 % CI: 21.0, 24.3) in 2019 to 24.9 (95 % CI: 23.2, 26.6) in 2022 but remaining stable throughout. (Table 1, Fig. 3, Fig. 4, Supplementary Table 1).

Black or African Americans consistently had higher prevalence rates than other racial groups, peaking at 44.5 (95 % CI: 42.3, 46.8) in 2020. Asian adults had the lowest rates but witnessed a non-significant increase from 10.3 (95 % CI: 8.5, 12.2) in 2019 to 12.6 (95 % CI: 10.8, 14.5) in 2022. A significant increase in the White population was noted from 31.9 (95 % CI: 31.1, 32.6) to 32.6 (95 % CI: 31.8, 33.5). Hispanic or Latino showed an increase from 35.8 (95 % CI: 33.9, 37.6) in 2019 to 37.6 (95 % CI: 35.7, 39.4) in 2022 which was again insignificant on trend analysis.

Obesity prevalence varied by MSA, with non-MSA areas showing the highest prevalence, rising from 39.7 (95 % CI: 37.8, 41.7) in 2019 to 40.2 (95 % CI: 38.1, 42.3) in 2022 while large MSA had the lowest rates. South and the Midwest had higher rates as compared to the Northeast and the West. Geographically, all census regions and MSAs showed no significant change in obesity when APCs were calculated.

Higher social vulnerability correlated with greater obesity rates decreasing slightly from 35 (95 % CI: 33.5, 36.5) to 34.1 (95 % CI: 32.8, 35.4), while little to no vulnerability populations had the lowest rates with both groups showing insignificant changes. Additionally, those with low social vulnerability showed a notable increase from 30.4 (29.1, 31.8) to 32.7 (31.3, 34.2) which gained significance on analysis of trends over four years. Employed individuals had consistently higher rates than non-employed individuals with employed individuals showing a significant increase in obesity over the years. US-born and veterans had consistently higher rates than foreign-born individuals and non-veterans. However, on analysis U.S-born and non-veterans had increasing rates of obesity that attained significance.

### 3.3 Annual trends and disparities in the prevalence of cigarette smoking among U.S. Adults

Cigarette smoking among U.S. adults aged 18 and over decreased insignificantly from 14 (95 % CI: 3.5, 14.5) to 11.6 (95 % CI: 11.1, 12.1) across the study period [APC: −6.2702 (95 % CI: −12.4023 to 0.4372)]. Male adults consistently had higher rates than females with both genders showing a decrease from 15.3 (95 % CI: 14.6, 16.1) to 13.2 (95 % CI: 12.5, 14.0) and 12.7 (95 % CI: 12.1, 13.4) to 10 (95 % CI: 9.4, 10.7) respectively with changes in males reaching significance on analysis [male APC: −5.0336∗ (95 % CI: −9.156 to −0.6731); female APC: −7.7106 (95 % CI: −15.1593 to 0.6526)].

**Fig. 5:**
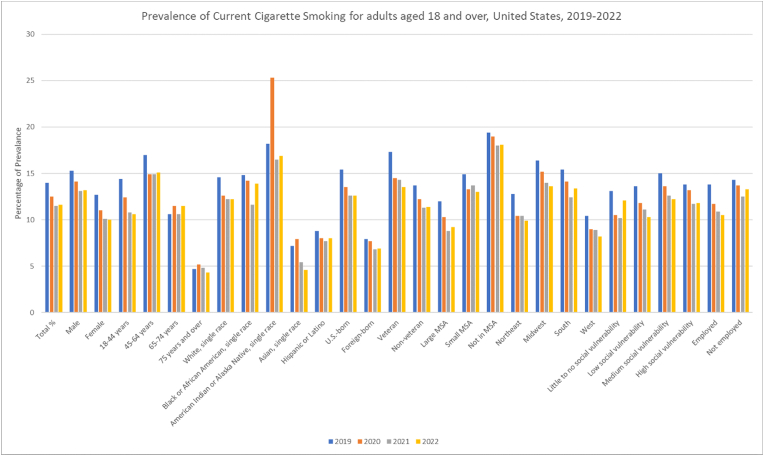
Prevalence of Cigarette Smoking for adults 18 and over in the United States 2019–2022.

**Fig. 6:**
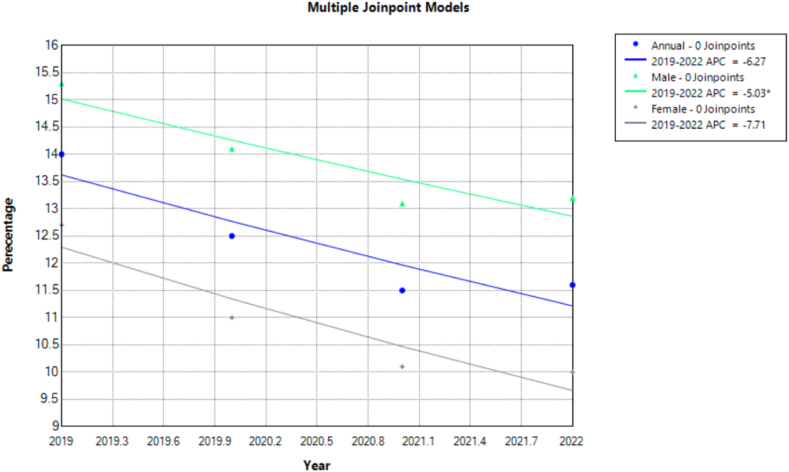
Annual Percentage Change of Cigarette Smoking for adults 18 and over in the United States 2019–2022.

Smoking was highest amongst adults aged 45–64 years at 17 (95 % CI: 16.1, 17.9) in 2019. Additionally, a sharp decline from 14.4 (95 % CI: 13.7, 15.2) to 10.6 (95 % CI: 9.9, 11.3) was witnessed in those between 18 and 44 years that reached significance on analysis. (Table 1, Fig. 5, Fig. 6, Supplementary Table 1).

Stratifying according to race, American Indians or Alaska Natives had consistently highest rates peaking at 25.3 (95 % CI: 17.6, 34.3) in 2021. Asians had the lowest rates falling significantly between 7.2 (95 % CI: 5.6, 9.0) and 4.6 (95 % CI: 3.5, 6.0) on analysis. Furthermore, Whites showed an apparent decrease from 14.6 (95 % CI: 14.1, 15.2) to 12.2 (95 % CI: 11.6, 12.7) which was insignificant.

Variations according to MSA revealed non-MSA showing the highest prevalence, declining from 19.4 (95 % CI: 18.0, 20.9) in 2019 to 18.1 (95 % CI: 16.4, 20.0) in 2022 with large MSA areas having the lowest. Respondents living in MSA and non-MSA areas all showed a significant decline in smoking rates when trends were analyzed. The Midwest regions had the highest rates while the West had the lowest with the gap significantly widening from 16.4 (95 % CI: 15.2, 17.6) to 13.6 (95 % CI: 12.5, 14.7) and 10.4 (95 % CI: 9.5, 11.3) to 8.2 (95 % CI: 7.3, 9.2) respectively from 2019 to 2022. Furthermore, a similar significant decrease in prevalence was also noted in residents of the Northeast.

Those with medium social vulnerability were found to have higher rates of smoking with prevalence decreasing from 15 (95 % CI: 14.0, 15.9) in 2019 to 12.2 (95 % CI: 11.2, 13.2) in 2022. Social vulnerability status did not impact smoking prevalence over the study period on statistical testing. Employed individuals generally had lower prevalence rates than the unemployed with both groups having stable prevalence on analysis. US-born had nearly double the rates of smoking than foreign-born with both populations showing decreases in rates from 15.4 (95 % CI: 14.8, 16.0) to 12.6 (95 % CI:12.1, 13.2) and 7.9 (95 % CI: 7.0, 8.9) to 6.9 (95 % CI: 6.0, 7.8) respectively. However, on annual trend analysis significance was only seen with the foreign-born subgroup Veterans had strikingly higher prevalence rates than non-veterans, though it decreased significantly from 17.3 (95 % CI: 15.5, 19.1) in 2019 to 13.5 (95 % CI: 11.9, 15.2) in 2022, still higher than the 13.7 (95 % CI: 13.2, 14.2) to 11.4 (95 % CI: 10.9, 11.9) for non-veterans which remained stable.

### 3.4 Annual trends and disparities in the Prevalence of High Cholesterol among U.S. Adults

**Fig. 7:**
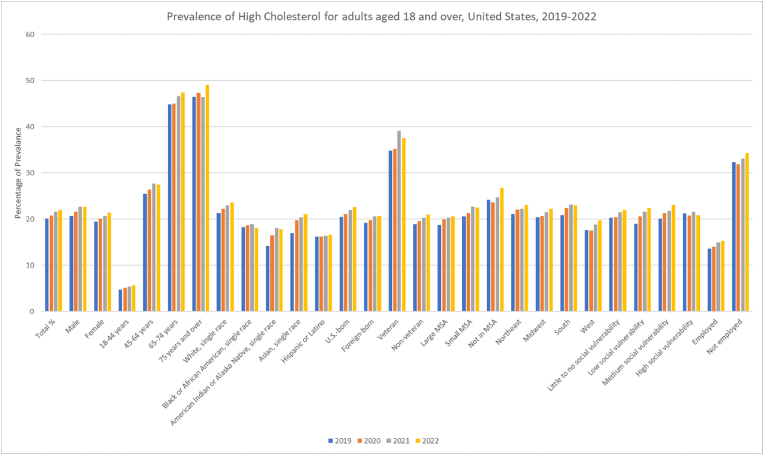
Prevalence of High Cholesterol for adults 18 and over in the United States 2019–2022.

**Fig. 8:**
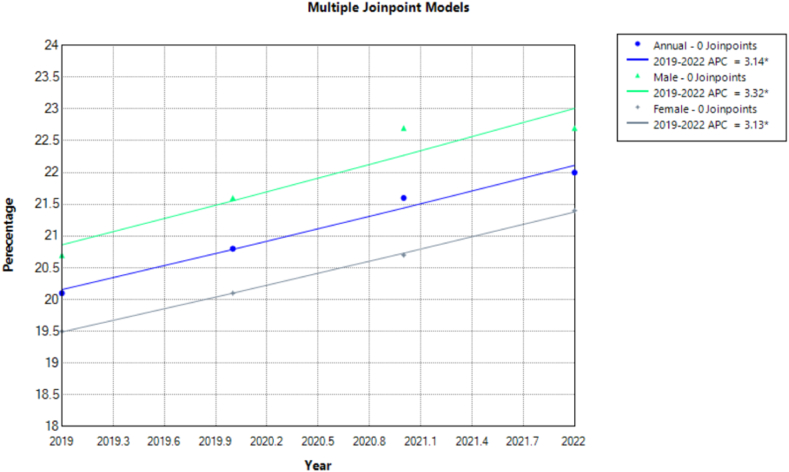
Annual Percentage Change of High Cholesterol for adults 18 and over in the United States 2019–2022.

Among U.S. adults aged 18 and over, the prevalence of High Cholesterol increased steadily from 20.1 (95 % CI: 19.6, 20.6) in 2019 to 22 (95 % CI: 21.5, 22.6) in 2022. Male adults consistently had a higher prevalence than females, rising from 20.7 (95 % CI: 19.9, 21.4) in 2019 to 22.7 (95 % CI: 21.8, 23.5) in 2022 while female prevalence ranged from 19.5 (95 % CI: 18.8, 20.2) in 2019 to 21.4 (95 % CI: 20.7, 22.2) in 2022. Annual trend analysis showed significant increases overall [APC: 3.1352∗ (95 % CI: 2.3273 to 3.9453)] among males [APC: 3.3175∗ (95 % CI: 1.1417 to 5.5416)] and females [APC: 3.1315∗ (95 % CI: 3.0191 to 3.2428)]. Increases with age were seen as higher rates in those aged 65–74 years and 75 years and over, peaking at 49.1 (95 % CI: 47.3, 51.0) for those 75 and older in 2022. All age groups younger than 75 years showed a significant increase in high cholesterol prevalence on analysis. (Table 1, Fig. 7, Fig. 8, Supplementary Table 1).

White adults consistently had higher prevalence rates than other racial groups, increasing significantly from 21.3 (95 % CI: 20.7, 21.9) in 2019 to 23.6 (95 % CI: 22.9, 24.3) in 2022 while the Hispanic or Latino population had the lowest rates also increasing significantly between 16.2 (95 % CI: 14.9, 17.6) and 16.6 (95 % CI: 15.3, 17.9) throughout the investigated period. Asian adults witnessed a significant increase from 17 (95 % CI: 15.0, 19.1) in 2019 to 21.1 (95 % CI: 18.9, 23.5) in 2022.

High Cholesterol levels significantly varied by MSA with non-MSA areas showing the highest prevalence, rising from 24.2 (95 % CI: 22.7, 25.8) in 2019 to 26.8 (95 % CI: 25.2, 28.4) in 2022 while those in large MSA had the lowest. Prevalence rates increased significantly over the years in MSA (large and small) areas. The West had the lowest rates of high cholesterol albeit showing an increase from 17.6 (95 % CI: 16.6, 18.7) to 19.7 (95 % CI: 18.6, 20.8) while the South and Northeast contrastingly had the highest rates.

Variations in high cholesterol prevalence were noted when stratifying patients according to social vulnerability status. All classes showed increases in rates except for those with high social vulnerability which showed a decrease from 21.2 (95 % CI: 20.0, 22.4) to 20.9 (95 % CI: 19.9, 22.0). None of these changes were significant on analysis. Employed individuals generally had half the rates than the unemployed, from 13.6 (95 % CI: 13.0, 14.2) in 2019 to 15.3 (95 % CI: 14.7, 16.0) in 2022, versus 32.3 (95 % CI: 31.2, 33.3) to 34.3 (95 % CI: 33.3, 35.4) for the unemployed both having no significant overall change. Veterans had higher prevalence rates than non-veterans, increasing insignificantly from 34.8 (95 % CI: 32.7, 36.9) in 2019 to 37.5 (95 % CI: 35.4, 39.7) in 2022, still higher than the 18.9 (95 % CI: 18.4, 19.4) to 21 (95 % CI: 20.4, 21.6) for non-veterans who showed a significant increase with US-born individuals also showing higher rates than those born abroad. Both nationalities noted statistically significant increases in prevalence on annual analysis.

### 3.5 Annual trends and disparities in the prevalence of arterial hypertension among U.S. Adults

Among the four risk factors analyzed, arterial hypertension is both a risk factor and cardiovascular disease, had the highest prevalence of about 27 % during the study period The prevalence of hypertension among males increased significantly from 27.2 % (95 % CI: 26.3, 28.1) in 2019 to 27.9 % (95 % CI: 27.0–28.8) in 2022 (APC 1.0234 %, 95 % CI: 0.2471 %–1.7943 %). Females presented a nonsignificant decrease from 26.9 % (95 % CI: 26.1–27.8) to 26.5 % (95 % CI: 25.7–27.3) across the study period (APC -0.4865 %, 95 % CI: −3.0964 %–2.2713 %). Prevalence increased significantly with age, peaking at 64.5 % (95 % CI: 62.1–66.3) for those 75 and older in 2019 (Supplementary Fig. 2). Black or African Americans consistently had the highest prevalence followed by Whites. Asian adults witnessed an increase in hypertension from 19.7 % in 2019 to 21.1 % in 2022. Additionally, Hispanic or Latino populations showed slight decreases from 20.2 % in 2019 to 19.7 % in 2022.

Prevalence also varied by metropolitan statistical areas with non-MSA areas showed a noticeably higher prevalence. Amongst the census regions, the South region had the highest prevalence of 30.2 % (95 % CI: 29.1–31.2) while the West had the lowest rates. Higher social vulnerability correlated with higher hypertension prevalence. The unemployed population had significantly increased rates of hypertension with 42.9 % (95 % CI: 41.9–44.0) as compared to 18.2 % (95 % CI: 17.5–18.9) in 2022 for the employed. Veterans’ status also showed higher prevalence of hypertension. Foreign-born individuals consistently reported lower prevalence rates compared to U.S.-born.

## 4 Discussion

This is the first study to assess the trends and disparities in the prevalence of circulatory disease risk factors in the US adult population, using nationally representative data. The main results of this study demonstrate that obesity was the most prevalent risk factor with higher APCs in females and higher prevalence rates in Black or African American adults and Southern and Midwestern regions of the country. Obesity was observed to have significantly increased in the age group 45–64 years during the study period. On the contrary, the prevalence rates of hyperlipidemia, the second most frequent cardiovascular disease risk factor, were higher among males, Whites, and the South and Northeast region. The hyperlipidemia prevalence trends demonstrated a significant increase across all age groups younger than 75 years. The diabetes prevalence also increased during the study periods, with males, Black or African American adults, and the Southern region having the highest prevalence rates for diabetes. The prevalence of cigarette smoking was found to decline during 2019–2022, however, the prevalence rates were higher amongst males, American Indians or Alaska Natives, and the Midwest region. Notably, a significant decline in the prevalence of cigarette smoking was observed in individuals aged 18–44 years over the study period.

In this NHIS database analysis, we found that an important modifier of the prevalence trends in circulatory disease risk factors was social vulnerability status. While increasing social vulnerability status corresponded to increasing diabetes and obesity prevalence, high cigarette smoking and hyperlipidemia prevalence were associated with medium social vulnerability status and low and medium social vulnerability status, respectively. Consistent with the findings of our analysis, employed individuals demonstrated a significant increase in the prevalence of obesity over the study period [15]. According to Yu et al., the high social vulnerability index of African Americans, Hispanics, women, and residents with a lack of healthcare insurance is linked to a greater percentage of fast-food restaurants and lesser access to exercise, linking the high social vulnerability index to individual-level and regional obesity [16]. In Europe, a higher prevalence of obesity was demonstrated in Roma and Latin American groups with no statistically significant association between belonging to ethnic minority groups and an increased prevalence of obesity, however, the higher prevalence of obesity was associated with the accumulation of more social vulnerabilities [17]. Notably, the increase in the prevalence of hyperlipidemia, obesity, and diabetes during 2019–2022 can be attributed to the exacerbation of social vulnerability during the COVID-19 pandemic, particularly in terms of race, housing, and economics [18,19].

Compared to high smoking rates in several lower-to-middle-income countries, the US and other economically developed countries have witnessed a decline in cigarette smoking in light of tobacco taxes, public awareness campaigns, and the implementation of other WHO Framework Convention for Tobacco Control measures [20]. Despite a consistent decline in the prevalence of cigarette smoking among adults and adolescents in the US, racial and gender-based disparities continue to exist. Consistent with the findings of our study, the prevalence of cigarette smoking was higher among American Indians and Alaskan Natives compared to Whites, according to the American Heart Association 2023 update [21]. The higher prevalence of smoking in the Midwest compared to other regions in the US can be attributed to several factors including prominent racial segregation in the region. Both Midwest and South regions exhibit limited smoking cessation treatments and availability of relevant resources, contributing to the rise in risk factor prevalence. Choi et al. also highlighted the prevalence of cigarette smoking was higher among Black or African American males and White females compared to their counterparts [22]. Our study reported the lowest rates of cigarette smoking among Asian Americans, which were statistically significant on analysis. A recent study, however, has demonstrated an increase in the current smoking rates among Asian Americans including Chinese and South Asians, which can be largely attributed to the COVID-19 pandemic and the associated exacerbation in the racial discrimination against this population [23]. This necessitates further exploration of prevalence rates of circulatory disease risk factors among ethnic minorities in the U.S. after adjusting for baseline demographics. Based on the findings of a pooled analysis of South Asian countries, factors strongly predictive of cigarette smoking were lower education status, occupation of any kind, lower economic status, and higher age [24]. Likewise, our study demonstrated lower prevalence rates of obesity among employed individuals compared to unemployed individuals.

Similar to the findings of our analysis during 2019–2022, the analysis of 2003–2018 trends in the US also highlighted that the prevalence of obesity was higher among females, non-Hispanic Blacks, and older adults ages 60–69 years. Compared to males, a significantly positive linear trend was observed in females for obesity as per 2005–2014 NHANES data. This can be explained by lower educational levels, the lesser likelihood of being physically active compared to their male counterparts, and estrogen-reducing postprandial fatty acid oxidation, which collectively contributes to obesity in females [25]. The higher prevalence rates of obesity among non-Hispanic Blacks can be explained by greater challenges encountered by this population in the implementation of healthy diets and physical activity, higher weight misperceptions, and preference for larger body sizes compared to non-Hispanic Whites. Notably, the poverty and unemployment rates are also greater in the non-Hispanic Black group [[26], [27], [28]]. Likewise, the prevalence of metabolic syndrome is higher among older adults in the Midwest and South, with significant association with the unemployed labor force in the Midwest and African American populations in the South [29,30].

Metabolic syndrome, characterized by the cluster of hypertension, hypertriglyceridemia, and hyperglycemia, is known to be associated with the development of cardiovascular diseases and diabetes mellitus. The prevalence of metabolic syndrome increased among older adults and decreased among populations with high socioeconomic status and household income. This is coherent with the findings of our study [31]. White males have a higher prevalence of hypertriglyceridemia among White males compared to Black males [32], however, Black adults have a higher prevalence of diabetes [33]. Additionally, diabetes-related complications including lower extremity amputation are more frequent among residents of the Southern region [34]. Importantly, diabetes and obesity have a synergistic effect on adverse cardiovascular indicators and mortality risk, with a stronger correlation between adiposity metrics and cardiovascular biomarkers [35]. In our study, African American, males, and residents from the South region showed the highest prevalence of hypertension.

The circulatory disease risk factors analyzed in this study are also closely linked to the development of cardiovascular-kidney-metabolic (CKM) syndrome, a disorder with interconnected heart disease, kidney pathologies, obesity, and diabetes mellitus [36]. In the year 2021, CKM syndrome was identified as the leading cause of death in the U.S. During 2011–2020, approximately 90 % of the U.S adult population met the criteria of CKM syndrome stage 1 or higher, and almost 15 % of the adults had advanced stage CKM syndrome, with none of these patients’ demonstrating improvement during this period. Trends analyzed during the same period indicated greater prevalence of advanced CKM syndrome stages in adults aged ≥65 years, in Black adults, and males [37]. Early identification and management of CKM risk factors including dyslipidemia, hypertension, and diabetes mellitus is recommended to mitigate the disease burden and improve treatment-related outcomes [38].

This study has identified obesity as a significant risk factor for circulatory diseases. However, a global temporal trend analysis conducted by Wang et al. revealed that high systolic blood pressure and elevated levels of low-density lipoprotein cholesterol represent the most critical cardiovascular disease risk factors. Our findings align with this analysis, indicating that males exhibit a higher likelihood of being affected by all risk factors compared to females, predominantly for smoking [39]. Furthermore, an analysis of regional and temporal trends in cardiovascular disease risk factors across European countries indicates a greater likelihood of risk factor-cardiovascular disease associations in males. Conversely, individuals with diabetes mellitus in Southern Europe and Central Europe demonstrated a lower likelihood of such associations. Over the 30-year period from 1982 to 2012, minor reductions were observed in the hazards associated with non-high-density lipoprotein cholesterol and systolic blood pressure, while slight increases were noted in the hazard related to body mass index [40].

Additionally, our research identified a statistically significant increase in the prevalence of diagnosed diabetes among Asian Americans during the period from 2019 to 2022. This trend is consistent with a documented surge in the pooled prevalence of diabetes among South Asians, which rose from 11.29 % in the 2000–2004 period to 22.30 % during the 2020–2024 period. Notably, the prevalence of diabetes is higher among males and in urban areas [41]. Moreover, similar to White populations, Asians have exhibited a significant rise in the prevalence of hyperlipidemia throughout the study period. This trend is consistent with the heightened prevalence of coronary artery disease and related risk factors, including dyslipidemia, within the South Asian population. Contributing factors include dietary practices, such as the consumption of saturated fats, and a greater genetic predisposition among South Asians. These observations underscore the necessity for comprehensive risk evaluations for South Asian migrants [42].

In this analysis, the authors identified specific populations, racial entities, and regions experiencing higher increases in circulatory disease risk factors, which may aid in the development of targeted intervention and prevention programs. The findings of our study may inform the evaluation of current policies in mitigating the burden of risk factors and also facilitate resource allocation while addressing the existing disparities. Future research and surveillance studies shall focus on exploring the social, economic, and environmental determinants of disparities. Additionally, longitudinal studies are a useful approach to monitor the prevalence trends and emerging risk factors and assess the outcomes of targeted interventions. Lastly, healthcare practitioners and other stakeholders may work towards raising public awareness through awareness campaigns, implementing health equity initiatives, and developing community-based programs to promote healthy living, strengthen tobacco control policies, and enhance screening and management of hypertension.

### 4.1 Limitations

In this study, the authors have analyzed and thoroughly described the prevalence trends and disparities of each of the four circulatory disease risk factors among US adults from 2019 to 2022. However, this study has several limitations. This retrospective analysis of the prevalence trends and disparities limits the determination of causal associations of circulatory disease risk factors or track changes in the prevalence trends over time given the cross-sectional nature of the survey. Next, the findings of this database analysis may not be generalizable to specific populations and settings across the globe. Consistent with the prior discussion, the COVID-19 pandemic may have exacerbated the existing disparities in the prevalence trends of certain risk factors, which haven't been taken into account. Lastly, during the COVID-19 pandemic, the NHIS data collection predominantly relied on telephone-based surveys, with regular interviewing procedures resuming in May 2021. A modified data collection approach may have influenced the observed estimates during the pandemic period.

## 5 Conclusion

In this study, we investigated the prevalence trends and disparities in four circulatory disease risk factors including obesity, diabetes mellitus, hyperlipidemia, and cigarette smoking among US adults during the period 2019–2022. According to the NHIS database analysis, obesity and hyperlipidemia were the two most prevalent risk factors for circulatory diseases in the country. Besides obesity having a higher female prevalence, males had a higher prevalence of diabetes, hyperlipidemia, and cigarette smoking during the study period. Our analysis also highlighted significant regional and racial disparities in the prevalence of circulatory disease risk factors, emphasizing the development of targeted public health interventions for mitigating the burden of circulatory diseases in the country.

## CRediT authorship contribution statement

**Farah Yasmin:** Writing – original draft, Data curation, Conceptualization. **Abdul Moeed:** Writing – original draft, Data curation, Conceptualization. **Hafsah Alim Ur Rahman:** Writing – original draft, Resources, Methodology, Investigation. **Muhammad Ahmed Ali Fahim:** Writing – original draft, Software, Resources, Methodology, Investigation. **Afia Salman:** Writing – original draft, Validation, Software, Resources. **Maryam Shaharyar:** Writing – original draft, Data curation, Conceptualization. **Rohan Kumar Ochani:** Writing – original draft, Software, Investigation, Formal analysis. **Afsana Ansari Shaik:** Writing – review & editing, Software, Investigation. **Muhammad Sohaib Asghar:** Writing – review & editing, Validation, Supervision, Conceptualization. **M. Chadi Alraies:** Writing – review & editing, Visualization, Supervision, Project administration.

## Consent to participate

Not applicable.

## Consent to publish

Not applicable.

## Clinical trial number

not applicable.

## Human ethics and consent to participate approval

Not applicable.

## Data and materials

Data sharing is not applicable to this article as no datasets were generated or analyzed during the current study. Existing datasets were analyzed for this study.

## Funding

None to declare.

## Declaration of competing interests

The authors have no relevant financial or non-financial interests to disclose.
