# Supplementary material for: Trends and disparities in the prevalence of circulatory disease risk factors among U.S. adults from the National Health Interview Survey database (2019–2022)
Source: Int J Cardiol Cardiovasc Risk Prev. 2025 Mar 8;25:200393. doi: 10.1016/j.ijcrp.2025.200393 (PMC11951206; doi:10.1016/j.ijcrp.2025.200393)

**Trends and disparities in the prevalence of circulatory disease risk factors among US adults from 2019 to 2022**

| **Variable** | **Diagnosed Diabetes** | **Obesity** | **High Cholesterol** | **Cigarette smoking** |
| --- | --- | --- | --- | --- |
| **Overall** | 1.278 (-0.296 to 2.8866) | 0.8632 (-0.0334 to 1.7804) | 3.1352* (2.3273 to 3.9453) | -6.2702 (-12.4023 to 0.4372) |
| **Male** | 2.0573 (-0.9107 to 5.0808) | 0.6185* (0.0422 to 1.2038) | 3.3175* (1.1417 to 5.5416) | -5.0336* (-9.156 to -0.6731) |
| **Female** | -0.4266 (-3.0439 to 2.2794) | 1.167 (-0.2284 to 2.5773) | 3.1315* (3.0191 to 3.2428) | -7.7106 (-15.1593 to 0.6526) |
| **18-44 years** | -0.4247 (-2.4003 to 1.5515) | 0.5925 (-1.5628 to 2.7785) | 6.5652* (4.9516 to 8.2286) | -10.0330* (-15.681 to -3.9377) |
| **45-64 years** | 2.3864 (-7.5938 to 13.491) | 1.2620* (0.2657 to 2.2701) | 2.7840* (0.7181 to 4.8753) | -3.4931 (-10.2165 to 3.8223) |
| **65-74 years** | -0.4457 (-3.4886 to 2.7003) | 0.0912 (-3.8656 to 4.2261) | 2.0628* (1.3334 to 2.7873) | 1.6432 (-6.21 to 10.2072) |
| **75 years and over** | -1.7487 (-6.6741 to 3.3516) | 2.717 (-3.6502 to 9.5896) | 1.4505 (-0.3634 to 3.256) | -3.4094 (-14.1616 to 8.925) |
| **White, single race** | 1.0263 (-1.198 to 3.313) | 0.6533* (0.2435 to 1.0659) | 3.4897* (2.4826 to 4.5283) | -5.5502 (-12.8291 to 2.5128) |
| **Black or African American, single race** | 1.3754 (-3.6126 to 6.5712) | 1.481 (-5.0544 to 8.6061) | -0.1695 (-3.26 to 3.0461) | -3.8293 (-12.468 to 5.4371) |
| **American Indian or Alaska Native, single race** | -5.7544 (-22.6706 to 15.1471) | 0.6633 (-9.087 to 11.762) | 8.0088 (-2.532 to 19.9334) | -6.2911 (-23.8961 to 15.2336) |
| **Asian, single race** | 4.9226* (1.8891 to 8.075) | 7.0383 (-2.2082 to 17.4758) | 7.0697* (0.8135 to 13.7852) | -15.8403* (-25.7771 to -4.4999) |
| **Hispanic or Latino** | 2.4119 (-0.4387 to 5.3285) | 1.2123 (-1.0949 to 3.5781) | 0.8581* (0.3028 to 1.4242) | -3.1895 (-10.9488 to 5.5199) |
| **U.S.-born** | 0.8808 (-1.4466 to 3.2244) | 0.9598* (0.4052 to 1.5138) | 3.4000* (2.7841 to 4.0169) | -6.4899 (-14.1139 to 2.061) |
| **Foreign-born** | 3.2061 (-0.3913 to 6.945) | 0.1681 (-3.504 to 3.9875) | 2.7403* (1.6577 to 3.842) | -5.1651* (-8.1064 to -2.1773) |
| **Veteran** | -0.9865 (-7.069 to 5.4934) | 0.1093 (-4.2532 to 4.6798) | 3.3473 (-1.0239 to 7.9329) | -7.2993* (-11.2052 to -3.2965) |
| **Non-veteran** | 1.7991* (0.4608 to 3.144) | 0.8966* (0.1588 to 1.6494) | 3.5741* (3.408 to 3.745) | -6.0867 (-12.6842 to 1.1521) |
| **Large MSA** | 1.4054 (-0.8847 to 3.7411) | 1.3946 (-2.0051 to 4.9016) | 3.1507* (0.4804 to 5.933) | -9.1036* (-16.8514 to -0.5152) |
| **Small MSA** | 2.5885 (-0.3854 to 5.6623) | 0.6329 (-1.4295 to 2.727) | 3.3378* (1.3314 to 5.4051) | -3.7249* (-6.9054 to -0.4826) |
| **Not in MSA** | -1.981 (-4.4195 to 0.5166) | 0.5265 (-0.0577 to 1.1172) | 3.5796 (-2.7618 to 10.4877) | -2.5874* (-3.9359 to -1.24) |
| **Northeast** | 1.2882 (-0.244 to 2.8476) | -0.4784 (-2.3161 to 1.371) | 2.8004* (1.1668 to 4.4587) | -7.4178* (-12.6519 to -1.9242) |
| **Midwest** | 3.6310* (0.7341 to 6.5744) | 1.6659 (-0.4299 to 3.8679) | 2.9588* (2.3069 to 3.6095) | -6.2358* (-8.0411 to -4.3819) |
| **South** | -0.0921 (-1.0023 to 0.8301) | 0.4697 (-2.5142 to 3.5139) | 3.2758 (-1.7599 to 8.7103) | -5.3119 (-11.125 to 0.8805) |
| **West** | 0.7256 (-0.9169 to 2.3872) | 1.7555 (-0.6753 to 4.2057) | 4.1833* (2.2651 to 6.104) | -6.9859* (-9.8504 to -4.0919) |
| **Little to no social vulnerability** | 1.1434 (-0.5266 to 2.846) | 1.3787 (-0.8004 to 3.5725) | 2.9814* (1.9372 to 4.0181) | -2.6367 (-24.6082 to 26.6655) |
| **Low social vulnerability** | 3.6851 (-1.3176 to 8.975) | 2.0487* (0.3534 to 3.771) | 5.5618* (2.8362 to 8.4206) | -8.5606* (-10.9391 to -6.1053) |
| **Medium social vulnerability** | 0 (-2.6976 to 2.8541) | 0.1555 (-1.628 to 1.9645) | 4.5039* (2.7299 to 6.3194) | -6.7253* (-10.2662 to -2.9667) |
| **High social vulnerability** | -2.4019 (-9.2379 to 5.0021) | -0.2879 (-5.4648 to 5.1167) | -0.0501 (-2.5557 to 2.5263) | -5.7325* (-8.5549 to -2.8624) |
| **Employed** | 5.0191* (1.3767 to 8.8327) | 0.7582* (0.5608 to 0.9531) | 4.2441* (2.6085 to 5.9116) | -8.5219* (-14.8532 to -1.5782) |
| **Not employed** | -1.2884 (-4.516 to 2.0699) | 1.1939 (-0.8476 to 3.302) | 2.1954* (0.0057 to 4.4634) | -3.0442 (-6.5839 to 0.5674) |

**Supplementary Table 1.** Annual percentage change of Diagnosed Diabetes, High Cholesterol, Cigarette Smoking and Obesity for adults aged 18 and over, United States, 2019—2022 * indicates significance (α<0.05)

**Supplementary Figure 1.** Trends in Annual Percentage Change of Diagnosed Hypertension for adults 18 and over in the United States 2019-2022.


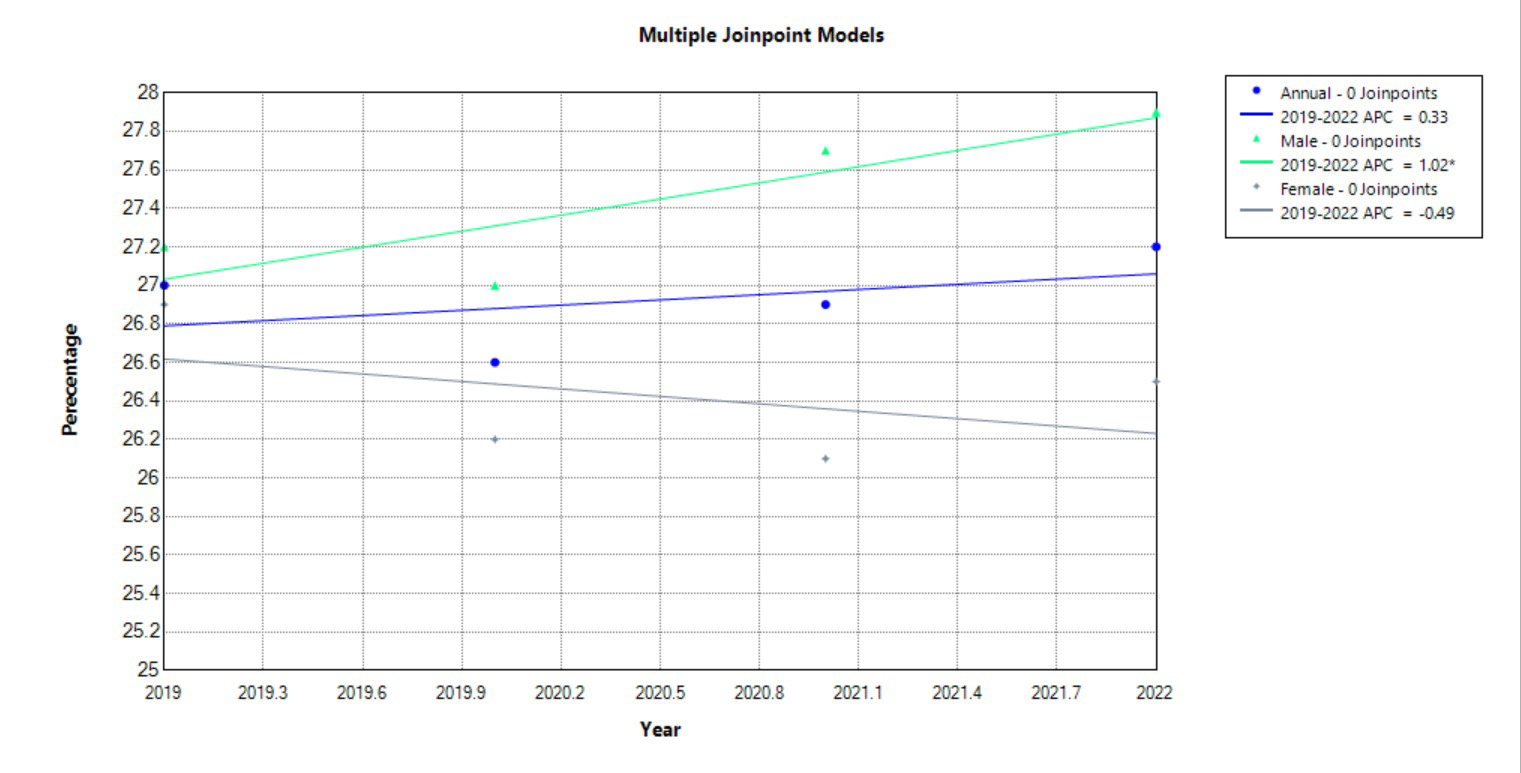


**Supplementary Figure 2. Prevalence of Diagnosed Hypertension for adults 18 and over in the United States 2019-2022.**


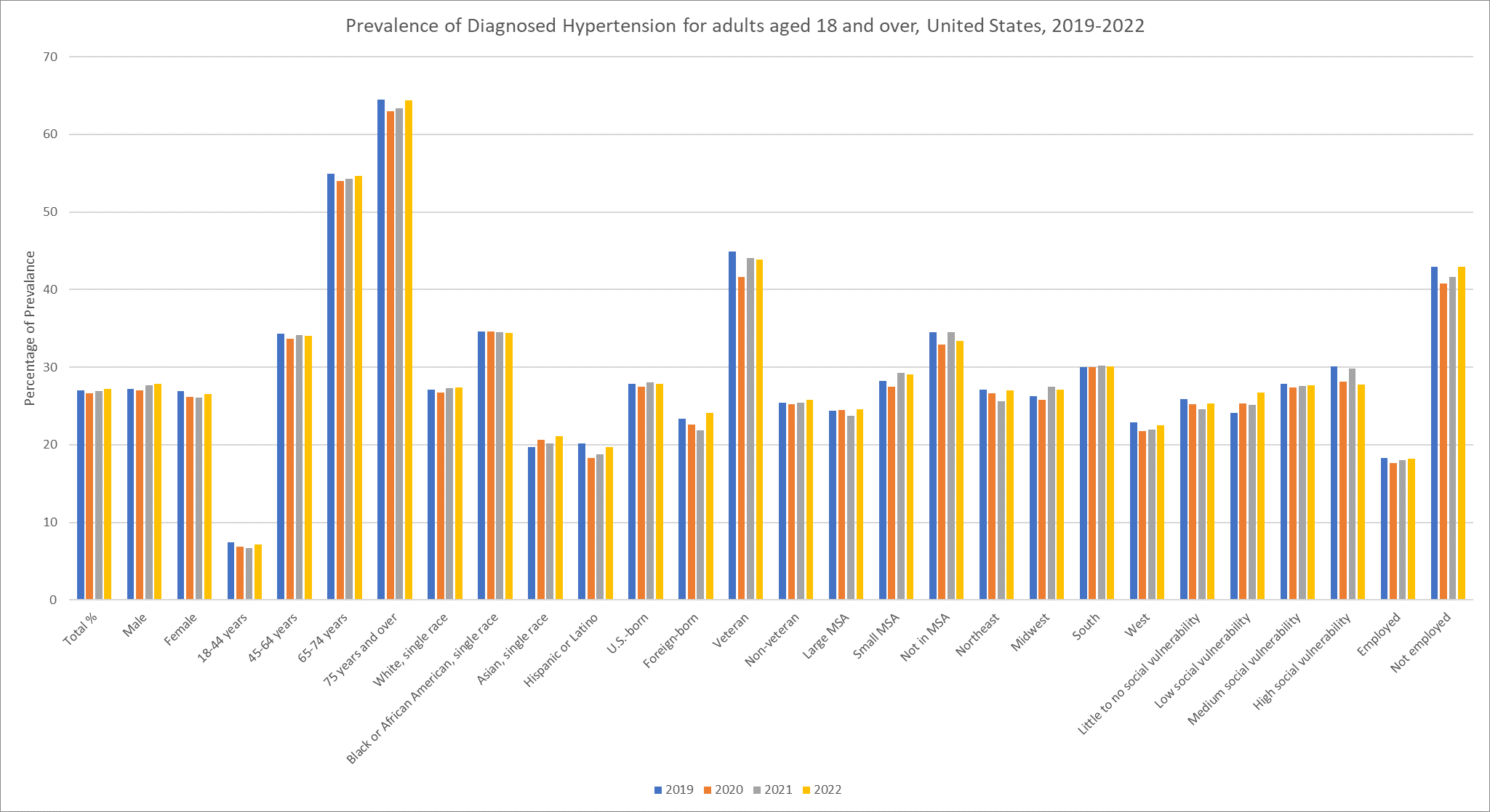

Supplement: Multimedia component 1 [file mmc1.docx]
